# Machine-Learning Approach to Identify Tissue Inhibitors of Metalloproteinases (TIMP) and Clinical Variables Predicting Executive Phenotypes in HIV-infected Adults

**DOI:** 10.1093/ofid/ofag335

**Published:** 2026-06-03

**Authors:** Ya-Wei Weng, Hung-Chin Tsai, Susan Shin-Jung Lee, Chih-Hui Hsu, Sheng-Hsiang Lin

**Affiliations:** Institute of Clinical Medicine, College of Medicine, National Cheng Kung University, Tainan, Taiwan; Division of Infectious Disease, Department of Internal Medicine, Kaohsiung Veterans General Hospital, Kaohsiung, Taiwan; Division of Infectious Disease, Department of Internal Medicine, Kaohsiung Veterans General Hospital, Kaohsiung, Taiwan; Faculty of Medicine, School of Medicine, National Yang Ming Chiao Tung University, Taipei, Taiwan; School of Medicine, College of Medicine, National Sun Yat-Sen University, Kaohsiung, Taiwan; Institute of Biomedical Sciences, National Sun Yat-Sen University, Kaohsiung, Taiwan; Division of Infectious Disease, Department of Internal Medicine, Kaohsiung Veterans General Hospital, Kaohsiung, Taiwan; Faculty of Medicine, School of Medicine, National Yang Ming Chiao Tung University, Taipei, Taiwan; School of Medicine, College of Medicine, National Sun Yat-Sen University, Kaohsiung, Taiwan; Biostatistics Consulting Center, National Cheng Kung University Hospital, College of Medicine, National Cheng Kung University, Tainan, Taiwan; Institute of Clinical Medicine, College of Medicine, National Cheng Kung University, Tainan, Taiwan; Biostatistics Consulting Center, National Cheng Kung University Hospital, College of Medicine, National Cheng Kung University, Tainan, Taiwan; Department of Public Health, College of Medicine, National Cheng Kung University, Tainan, Taiwan

**Keywords:** executive function, HIV, machine learning, TIMP

## Abstract

**Background:**

Despite effective combination antiretroviral therapy (cART), executive function impairment remains prevalent among people living with HIV (PLWH). The pathogenesis of HIV-associated neurocognitive disorder is multifactorial, involving chronic immune activation, metabolic alterations, and neuroinflammatory processes. Matrix metalloproteinases and their tissue inhibitors (TIMPs) contribute to neuroinflammation and blood–brain barrier disruption, but their relationship with executive dysfunction in HIV remains unclear.

**Methods:**

We enrolled 169 middle-aged Taiwanese PLWH who underwent comprehensive clinical and laboratory assessments. Executive function was evaluated using Wisconsin Card Sorting Test, and participants were classified into executive impaired and unimpaired group. Six variables—years since HIV diagnosis, BMI classification, nadir CD4 count, and plasma levels of TIMP-1, TIMP-2, and TIMP-4—were used to develop predictive models. Logistic regression, support vector machine, random forest, and Extreme Gradient Boosting (XGBoost) algorithms were applied. Model performance was evaluated using the area under the receiver operating characteristic curve with 10-fold cross-validation, and SHAP analysis was used to interpret feature contributions.

**Results:**

Executive impairment was identified in 9.5% of participants. Among single predictors, TIMP-1 demonstrated the highest discriminative performance (AUC = 0.76 by XGBoost), whereas TIMP-2 and TIMP-4 showed lower predictive performance. Combining clinical and laboratory variables markedly improved model performance, with XGBoost achieving an AUC of 0.89. Ten-fold cross-validation yielded a mean AUC of 0.77, supporting model robustness.

**Conclusions:**

Executive dysfunction remains an important neurocognitive phenotype in middle-aged PLWH. Integrating clinical and biological variables through machine-learning supports early identification of individuals at risk and may facilitate timely neuropsychological evaluation and intervention.

With the widespread use of combined antiretroviral therapy (cART), the life expectancy for people living with human immunodeficiency virus type 1 (HIV-1) has increased dramatically [1]. Though the overall life expectancy increased over time in individuals with HIV infection, there remains a gap in comorbidity-free years between individuals with HIV and those without HIV [2]. HIV is neuroinvasive with early involvement of nervous system and has a wide range of clinical presentations [3]. Though in the cART era, a decrease in the incidence of opportunistic infections of central nervous system (CNS) [4], HIV-associated neurocognitive disorder (HAND) remains prevalent [5, 6]. According to a previous systematic review, the overall global prevalence of HAND was 42.6% [7]. More effective therapies have reduced the severity of HAND, but the HAND remains prevalent in a milder form [8]. In these neurocognitive impairment populations, deficits in motor speed and speed of information processing were more predominant in pre-cART era, whereas the learning and executive function domains were more affected in the cART era [5]. In the cART era, a systematic review shows that performance decrements were found among patients living with HIV (PLWH) in all domains of executive function [9]. Another recent meta-analysis focused on older adults with HIV infection, which enrolled 21 cross-sectional studies, provided evidence that HIV infection is associated with an increased risk of executive function, motor/psychomotor, and processing speed among older adults [10]. The impairment of executive function is still an important issue in PLWH in the cART era.

The pathogenesis of HAND is multifactorial, including early HIV infection of the CNS, chronic viral replication in the CNS, chronic immune activation in the CNS, toxicity and/or penetration of cART, other comorbidities, substance abuse, etc [11]. Numerous clinical factors have been identified as linked to neurocognitive impairment in PLWH, including nadir CD4 count [12], length of HIV infection [13, 14], lack of virologic suppression [15], cardiovascular risk factors and diseases [14, 16], central obesity [13], and coinfection with hepatitis C virus [17].

Matrix metalloproteinases (MMPs) comprise a family of 23 zinc-dependent endopeptidases responsible for extracellular matrix turnover and remodeling. Among them, MMP-2 and MMP-9 are of particular relevance to the CNS, as they degrade components of the extracellular matrix and basement membrane, thereby increasing the permeability of the blood–brain barrier (BBB) [18]. Elevated expression of MMP-2 and MMP-9 has been associated with neuroinflammation and neurocognitive impairment [19–21]. Beyond their structural functions, MMPs are now recognized to participate in diverse biological processes, including cell signaling, immune modulation, and transcriptional regulation. In CNS disorders, MMPs often act as a double-edged sword—contributing to both neuronal injury and tissue repair depending on the disease context [22]. The activity of MMPs is tightly controlled by 4 endogenous tissue inhibitors of metalloproteinases (TIMPs). Among them, TIMP-2 primarily inhibits MMP-2, while TIMP-1 is the predominant inhibitor of MMP-9 [23]. TIMPs have been implicated in neuroprotection, particularly through the maintenance of BBB integrity. Dysregulation of the MMP/TIMP balance has been linked to various inflammatory and neurodegenerative diseases [24, 25]. However, whether TIMP-1 consistently exerts a protective role within the CNS remains unclear. Recent studies have revealed that TIMP-1 also functions as a signaling molecule in immune cells, influencing inflammatory responses beyond its canonical role as an MMP inhibitor. Elevated TIMP-1 levels have been reported in a wide range of inflammation-associated disorders and are often associated with poor clinical outcomes [26–28]. Given the multifaceted roles of MMPs and TIMPs in neuroinflammation and neuronal integrity, these molecules may serve as key mediators linking chronic immune activation to cognitive dysfunction in people living with HIV.

The rate of neurological disorders in HIV-positive adults in Taiwan is ∼13.67 per 1000 person-years [29]. Data regarding the cognitive abilities of patients with HIV infection in Taiwan remain limited. In one recent study using the Alzheimer's Disease-8 (AD8) questionnaire as a screening tool for cognitive impairment among PLWH in Taiwan, the reported prevalence was 2.25% [30]. However, the AD8 is a brief screening instrument and may have limited sensitivity for detecting mild or domain-specific neurocognitive impairment. In contrast, studies conducted in other regions that employ comprehensive neuropsychological assessments have reported substantially higher prevalence estimates of HAND, which ranges from 13.9% to 87% [31, 32]. Therefore, differences in assessment methodologies may contribute to the variability in reported prevalence, and the burden of cognitive impairment in Taiwanese PLWH may be underestimated.

Machine-learning techniques have been used for classification, which can help in revealing potential hidden dependencies between factors and outcomes [33]. Few studies have shown that clinical variables and blood biomarkers have the potential to inform patients with HIV infection likelihood of progression to executive impairment [15]. To gain a deeper insight into the executive function of PLWH in Taiwan, and to identify possible clinical factors and the specific contribution of TIMPs and MMPs that could predict the executive phenotype within populations with HIV, we conducted this research to build a prediction model. We applied machine-learning approaches to integrate clinical and laboratory parameters, aiming to identify predictive patterns associated with executive dysfunction among middle-aged individuals living with HIV.

## METHODS

### Study Population

This prospective cohort study was conducted at Kaohsiung Veterans General Hospital, which is one of the largest medical centers in Kaohsiung City with 1488 in-hospital beds. About 1500 PLWH were followed in this hospital regularly. Between November 1, 2020 and March 31, 2024, 169 patients with HIV infection who are more than 40 years old were enrolled for further executive function evaluation. All participants received standard HIV care in accordance with contemporary treatment guidelines, consistent with recommendations from the Taiwan Centers for Disease Control, the Taiwan AIDS Society [34], and established international guidelines. Participants were excluded if they had conditions that could independently affect cognitive function, including: (1) present or past history of CNS disease unrelated to HIV (eg, stroke, neurodegenerative disorders, or intracranial infections), (2) history of significant head trauma, or (3) known psychiatric disorders or current treatment with antipsychotic medications. At enrollment, the participants underwent clinical assessment, cognitive and depression screening tests, comprehensive executive function evaluation and laboratory tests. In addition to demographic characteristics, a comprehensive set of candidate clinical variables known or suspected to be associated with neurocognitive impairment in people living with HIV was collected. These included HIV-related factors (duration of HIV infection, baseline and nadir CD4 cell counts, HIV viral load at the time of assessment, and exposure to efavirenz), cardiovascular risk factors (body mass index [BMI] and smoking status), comorbid conditions (hepatitis B or C coinfection, syphilis, and psychiatric diagnoses), and substance use (alcohol or other substance dependence). The duration of HIV infection was defined as the time from the date of confirmed HIV diagnosis to study enrollment. Nadir CD4 count was defined as the lowest recorded CD4 cell count documented in the medical record prior to enrollment.

### Screening Tests

Complex neuropsychological assessments require a significant amount of time and resources, and the International HIV dementia scale (IHDS) is a brief 3-min screening test that is ideal for screening the frequency of cognitive impairment in newly referred patients [35–37]. The AD8 questionnaire is a short informant-based assessment designed to distinguish between cognitively healthy individuals and those with dementia [38, 39]. In the present study, the AD8 was administered as a self-reported questionnaire at the time of enrollment, and a cutoff score of ≥2 was used to indicate suspected cognitive impairment [38]. However, the sensitivity of these screening tools varies across different studies. Both of these screening tools are simple to administer and are commonly utilized as initial assessments for identifying individuals who might need formal neuropsychological evaluation.

### Neuropsychological Tests

Wisconsin Card Sorting Test (WCST) [40] is a widely used neuropsychological test for assessing executive functions [41, 42]. The WCST is a card matching task. WCST has been applied in Taiwanese and PLWH [43–45]. There are multiple parameters in the WCST, and several of them are commonly utilized to evaluate executive functions and cognitive flexibility, such as total errors, perseverative responses, perseverative errors, and conceptual level responses [42, 46–49]. These parameters are used as main outcomes.

### Enzyme-linked Immunosorbent Assay

Total protein concentrations of MMP-2, MMP-9, TIMP-1, TIMP-2, and TIMP-4 in plasma were measured using Enzyme-linked Immunosorbent Assay (ELISA) assays. The ELISA kits of MMP-2, MMP-9, TIMP-2, and TIMP-4 were purchased from R&D Systems (Minneapolis, MN). The ELISA kit of TIMP-1 was purchased from Sino Biological, Inc. (Beijing, China). ELISA results were documented using a microplate reader system (BioTek 800 TS Absorbance Reader, USA).

### Statistical Analysis

The adjusted z-scores of the WCST measures were calculated based on the normative data of 303 healthy participants, aged 21 to 65 years, who were recruited between 2011 and 2023 in southern Taiwan for establishing new norms. Adjustments were made for sex, age, and education. The computation followed the procedures described in previous studies [50, 51]. Specifically, for each subject, a predictive score was derived from the regression coefficients obtained from the normal comparison group, in which WCST scores were regressed on the demographic covariates. The difference between the subject's raw score and the predictive score was then standardized by the root mean square error of the regression model, yielding the adjusted z score. We used cluster analysis to identify clusters of participants with similar profiles as defined by 4 parameters from WCST. The cluster analysis uncovered clinical phenotypes associated with executive function, distinguishing between the executive unimpaired group and the executive-impaired group. Candidate variables were assessed based on demographic characteristics and established risk factors. Variable selection can reduce the complexity of the predictive model without much loss of the total information. It also helps to increase the interpretability and accuracy of the model. By using the χ2 test or Fisher's exact test for categorical variables and *t* tests or Mann–Whitney *U* test for continuous variables, depending on whether or not the continuous variable was normally distributed, to assess clinical and laboratory differences between the executive unimpaired and impaired groups. Clinical and laboratory variables with a *P*-value <.2 were enrolled for further predictive model building. Effect sizes for continuous variables were estimated using Cohen's *d*, whereas effect sizes for binary variables were derived from odds ratios using the Chinn conversion method [52].

Partial least squares-discriminant analysis (PLS-DA) was performed to visualize and classify the executive function phenotypes based on the combined clinical and laboratory variables. PLS-DA integrates features of PLS regression and linear discriminant analysis, enabling the modeling of data sets with a large number of collinear and intercorrelated predictors relative to the sample size [53]. This method projects the original variables into a lower-dimensional latent space that maximizes separation between executive phenotypes while preserving most of the explanatory variance in the data.

Prior to model development, multicollinearity among variables was assessed using variance inflation factors (VIF), with all variables demonstrating low collinearity (VIF < 2). Four machine-learning algorithms—Extreme Gradient Boosting (XGBoost), Logistic Regression (LR), Random Forest (RF), and Support Vector Machine (SVM)—were employed to predict the executive function phenotype. XGBoost, an optimized gradient-boosting framework, sequentially builds decision trees to minimize classification error and improve model generalization [54]. LR is a classical linear model used to estimate the probability of an outcome based on weighted input variables [55]. Random forest is an ensemble learning method that aggregates predictions from multiple decision trees to enhance accuracy and reduce overfitting [56]. Support vector machine constructs an optimal hyperplane to maximize the margin between classes, effectively handling nonlinear relationships through kernel functions [57]. The predictive performance of both single variables and combined markers was assessed by calculating the Area Under the Receiver Operating Characteristic Curve (ROC-AUC), providing a quantitative measure of the model's capability to discriminate between groups. We then assessed each model's combined markers using 10-fold cross-validation AUC to evaluate predictive performance. We performed SHAP (SHapley Additive exPlanations) analysis on the XGBoost model with the highest AUC to interpret the contribution and influence of each variable on the model's predictions. SHAP analysis is a method based on cooperative game theory that assigns each variable a value representing its contribution to the model prediction. The summary plot and decision plot allow us to understand which features most influence the model's output and how they impact individual predictions. All data were analyzed using Python version 3.12 and R software version 4.4.1.

## RESULTS

**Figure 1.**
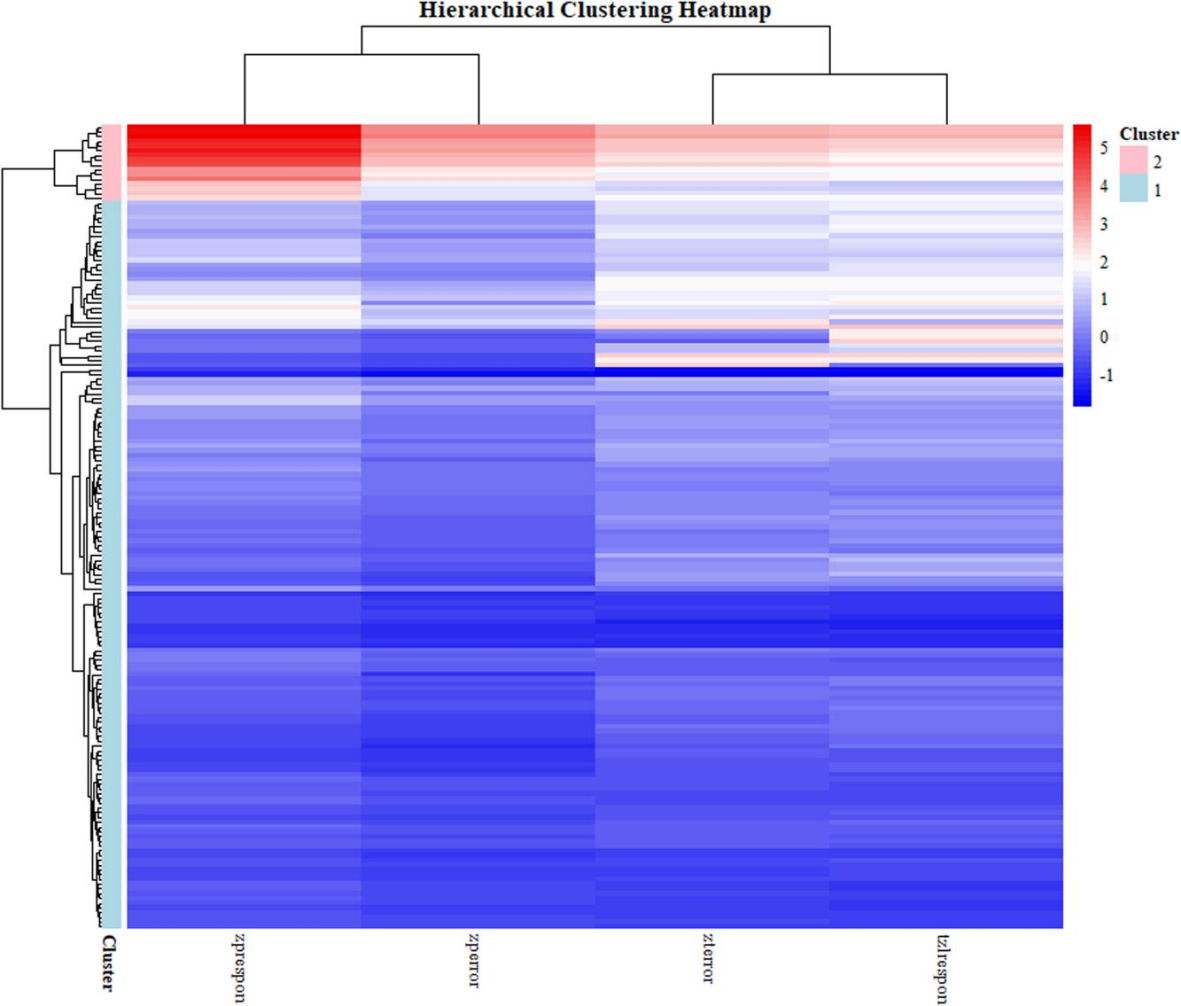
Cluster analysis based on 4 variables from Wisconsin Card Sorting Test (WCST), including total errors, perseverative response, perseverative errors, and conceptual level responses. Participants in cluster 1 were classified as executive unimpaired group (N = 153), participants in cluster 2 were classified as executive-impaired group (N = 16).

One hundred and sixty-nine participants were recruited, the majority of whom were man (n = 158, 93.5%). Their median age was 47 years. The median nadir CD4 count was 243/cumm (IQR, 98–389/cumm). Men who have sex with men accounted for 69% of the patient group. Most of them, 93%, were viral suppressed (<200 copies/ml) while receiving neuropsychological tests. Based on the AD8 questionnaire, 13% of participants had scores ≥2, indicating suspected cognitive impairment. IHDS scores ≤10 were observed in 56.8% of participants. Hierarchical cluster analysis based on 4 WCST-128 variables is presented in Figure 1, and the corresponding raw scores and adjusted z-scores are provided in Supplementary Table 1. The selection of 2 clusters, the executive unimpaired group and the executive-impaired group, was made due to the enhanced clarity of the outcomes. A total of 153 individuals are part of the executive unimpaired group, while 16 individuals are in the executive-impaired group. About 9.5% (16/169) of participants belong to the executive-impaired phenotype. The clinical characteristics and laboratory variables in both groups are shown in Table 1. By using the χ2 test or Fisher's exact test for categorical variables and *t* tests or Mann–Whitney *U* test for continuous variables. The *P*-values for years since HIV diagnosis, BMI classification (BMI < 27 vs BMI ≥ 27), nadir CD4 levels (CD4 ≥ 200 vs CD4 < 200), TIMP-1, TIMP-2, and TIMP-4 in both groups are all below .2, and these variables are included in the subsequent predictive model buildup.

**Table 1.** Clinical Characteristics and Laboratory Variables in Unimpaired and Impaired Executive Function Groups

	Unimpaired (N = 153)	Impaired (N = 16)	
Variables	n (%)/Median (IQR)	n (%)/Median (IQR)	*P*-value^a^
Clinical characteristics			
Age, median	47.00 (41.00, 52.00)	47.00 (42.50, 52.50)	.609
Male	143 (93.46)	15 (93.75)	1.000
Years since HIV diagnosis	10.00 (6.00, 15.00)	13.00 (9.00, 20.00)	.047
BMI (raw data)	24.11 (22.39, 26.26)	24.82 (21.71, 28.39)	.662
BMI class			.050
BMI < 27	123 (80.39)	9 (56.25)	
BMI ≥ 27	30 (19.61)	7 (43.75)	
Nadir CD4 (cells/μl)			.111
≥200	98 (64.05)	7 (43.75)	
<200	55 (35.95)	9 (56.25)	
Syphilis			.284
Negative	89 (58.17)	12 (75.00)	
Positive	64 (41.83)	4 (25.00)	
HCV Ab positive			.330
Negative	122 (80.26)	11 (68.75)	
Positive	30 (19.74)	5 (31.25)	
Route of HIV infection			.221
None	11 (7.19)	2 (12.50)	
MSM and bisexual	108 (70.59)	8 (50.00)	
IVDU	13 (8.50)	2 (12.50)	
Heterosexual	21 (13.73)	4 (25.00)	
EFV exposure >1 y			.761
No	110 (75.86)	11 (73.33)	
Yes	35 (24.14)	4 (26.67)	
HIV VL on performance date (copies/ml)			.570
<200	132 (93.62)	11 (91.67)	
≥200	9 (6.38)	1 (8.33)	
Screen test			
IHDS total			.962
>10	66 (43.14)	7 (43.75)	
≤10	87 (56.86)	9 (56.25)	
AD8			.442
≤2	134 (87.58)	13 (81.25)	
>2	19 (12.42)	3 (18.75)	
Laboratory			
MMP-9, ng/ml	319.21 (215.62, 492.70)	328.70 (211.13, 599.01)	.981
MMP-2, ng/ml	102.95 (83.35, 130.65)	105.27 (74.45, 121.51)	.774
TIMP-1, ng/ml	152.12 (125.92, 183.33)	176.78 (153.56, 253.66)	.015
TIMP-2, ng/ml	93.10 (80.53, 106.07)	102.39 (90.63, 109.14)	.107
TIMP-4, pg/ml	3431.37 (2481.40, 5011.68)	4403.71 (3512.14, 6183.40)	.044

Abbreviations: BMI, body mass index; HCV, hepatitis C virus; EFV, efavirenz; HIV, human immunodeficiency virus; IHDS, international HIV dementia scale; IVDU, intravenous drug users; MMP, matrix metalloproteinase; MSM, men who have sex with men; TIMP, tissue inhibitors of metalloproteinase; VL, viral load.

^a^Clinical and laboratory variables with a *P*-value <0.2 were enrolled for further predictive model building.

**Figure 2.**
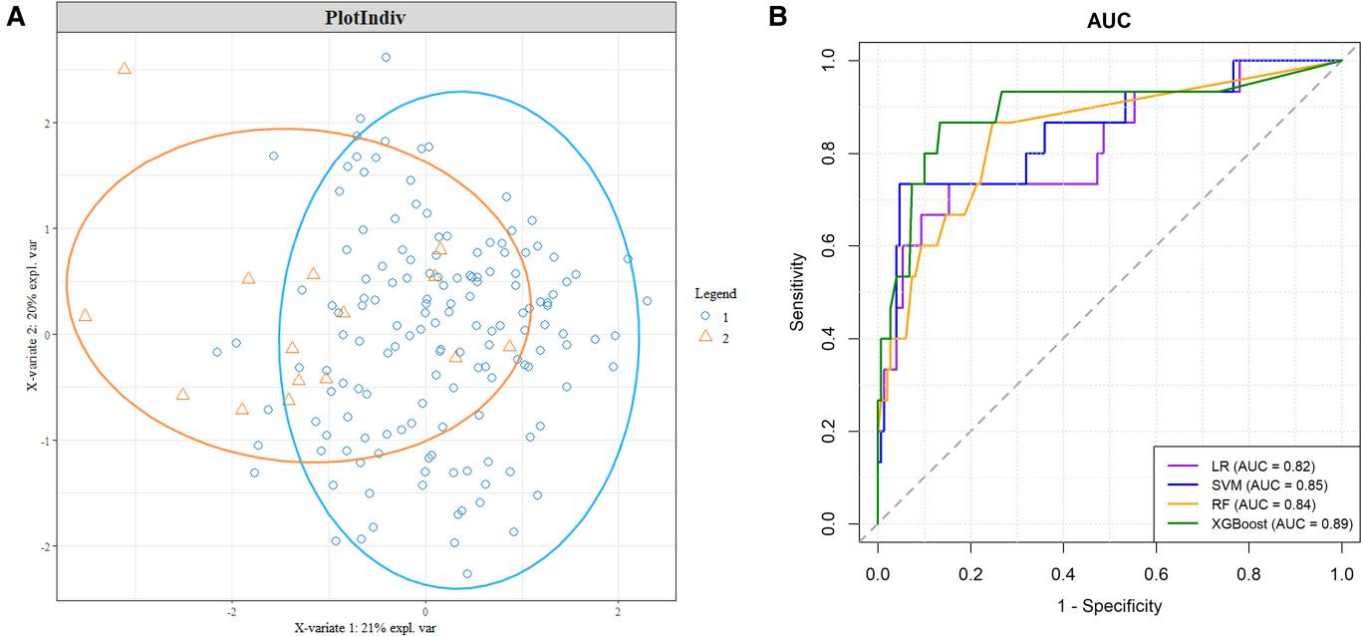
Discrimination between executive-impaired and unimpaired individuals living with HIV. *A*, Partial least squares-discriminant analysis (PLS-DA) model showing separate clustering of all samples from executive unimpaired group versus executive-impaired group. The correct classification rate is 73%. o executive unimpaired group; Δ executive-impaired group. *B*, Receiver operating characteristic (ROC) curves for estimating the discriminative ability of the logistic regression (LR) model, support vector machine model (SVM), random forest model (RF), and EXtreme Gradient Boosting model (Xgboost). AUC, area under the receiver operating characteristic curve.

To explore potential group separation between executive-impaired and unimpaired individuals, we first performed PLS-DA, as shown in Figure 2*A*. The latent variable score plot suggests a tendency toward separation between the 2 groups, with blue circles indicating unimpaired participants and red triangles indicating those with executive impairment, yielding a correct classification rate of 73%. To further quantify discriminative performance, 6 variables—years since HIV diagnosis, BMI classification (BMI < 27 vs ≥27), nadir CD4 levels (CD4 ≥ 200 vs <200), TIMP-1, TIMP-2, and TIMP-4—were incorporated into 4 machine-learning models (XGBoost, LR, random forest, and support vector machine). The ROC curves for each algorithm (Figure 2*B*) demonstrate their respective abilities to distinguish executive-impaired from unimpaired individuals. The XGBoost model had the highest AUCs for executive-impaired phenotype, with an AUC of 0.89. The AUCs of each variable in different machine-learning models are shown in Table 2. For each model, the 10-fold validation AUCs are also shown in Table 2.

**Table 2.** The AUC of Different Predictive Models for Impaired Executive function

Single Marker	LR	SVM	RF	XGBoost
TIMP-1	0.69	0.74	0.71	0.76
TIMP-2	0.62	0.65	0.63	0.70
TIMP-4	0.65	0.69	0.68	0.74
BMI	0.62	0.63	0.63	0.63
CD4	0.60	0.63	0.63	0.63
Years since HIV diagnosis	0.66	0.69	0.67	0.75
Combine markers	0.82	0.85	0.84	0.89
Ten-fold cross-validation	0.73	0.76	0.75	0.77

Abbreviations: AUC, area under the receiver operating characteristic curve; BMI, body mass index; HIV, human immunodeficiency virus; LR, logistic regression; RF, random forest; SVM, support vector machine; TIMP, tissue inhibitors of metalloproteinase; XGBoost, extreme gradient boosting.

**Figure 3.**
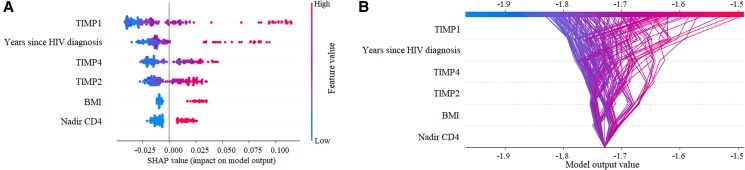
SHAP-based interpretation of the XGBoost model for predicting executive impairment. *A*, Shapley additive explanations (SHAP) summary plots showing SHAP values from all data points. The SHAP value indicates the contribution of each variable to the model's prediction, with positive values suggesting that the variable increases the likelihood of executive impairment, and negative values indicating a decrease. The color of the dots, ranging from blue to red, represents the variable's value, with blue corresponding to lower values and red indicating higher values of the predictor. BMI and nadir CD4 are binary variable. Nadir CD4 ≥ 200 is equal to 0, shown in blue; nadir CD4 < 200 is equal to 1, shown in red. BMI < 27 is equal to 0, shown in blue; BMI ≥ 27 is equal to 1, shown in red. *B*, SHAP decision plot demonstrating the decision path taken for each variable release prediction in the dataset. TIMP, tissue inhibitors of metalloproteinase; HIV, human immunodeficiency virus; BMI, body mass index.

As shown in Figure 3*A*, we used SHAP values of XGBoost to determine the importance of the above 6 variables for predicting executive phenotype. The x-axis shows the SHAP value of the variable, which can reflect the influence of the variable on the model, and is displayed in different colors, where red represents the high-risk value and blue represents the low risk value. The y-axis shows the variable value, which can reflect the predictive value of each predictor to the model. Figure 3*B* is the SHAP decision plot, which shows how variables cumulatively impact each patient's executive risk, helping prioritize high-risk patients for tailored interventions.

## DISCUSSION

In the present study, executive impairment was identified using the WCST, with a prevalence of 9.5% among middle-aged people living with HIV in Taiwan. Previous studies conducted in other countries have reported executive dysfunction rates ranging from approximately 20.2% to 47.8% among people living with HIV [44, 58]. In the study by Kanmogne et al., impairment in the executive function domain was observed in 20.2% of HIV-positive participants using a combination of executive function measures, including the WCST, Color Trails Test-II, and Stroop Color–Word Test [44]. In the same study, WCST-defined impairment based on total errors was observed in 25.6% of participants [44]. Another study by Shah et al. reported executive impairment in 47.8% of asymptomatic HIV-positive individuals using WCST-based classifications [58]. However, direct comparison across studies remains difficult because of substantial methodological heterogeneity. In particular, prior studies using the WCST often defined impairment using single outcome measures such as total errors, whereas our study incorporated multiple WCST-derived indices to characterize executive phenotypes. Differences in cutoff thresholds, normative reference standards, and combinations of neuropsychological instruments may further contribute to variability in reported prevalence. In addition, WCST performance in our study was standardized using general population norms rather than HIV-specific reference data, which may influence interpretation of executive function impairment in people living with HIV. Systematic reviews have shown that patients with HIV infection are more prone to executive dysfunction than those who are HIV-negative [9]. Our findings are consistent with previous studies that executive dysfunction is still prevalent in the era of cART. The relatively lower prevalence observed in our cohort may reflect differences in assessment methodologies and study populations.

Wu HC et al [30] reported a prevalence of 2.25% for cognitive impairment among HIV-positive adults in Taiwan using the AD8 questionnaire (cutoff ≥2). Given that the AD8 is a brief screening tool rather than a domain-specific neuropsychological assessment, direct comparison with WCST-based executive function outcomes is limited. In our study, a higher proportion of participants had AD8 scores ≥2 (13%), suggesting that screening instruments and performance-based assessments may capture different aspects of cognitive function. The discrepancy between studies may also be partly explained by differences in participant characteristics, as our cohort consisted of older individuals with a longer duration of HIV infection—both factors associated with increased risk of cognitive impairment. Furthermore, the lack of a significant association between AD8 scores and WCST-defined executive impairment in our cohort (Table 1; *P* = .442) suggests that the AD8 may have limited sensitivity for detecting domain-specific executive dysfunction.

The use of the IHDS as a screening tool warrants further consideration. Although the IHDS is a rapid and practical instrument for detecting HIV-associated neurocognitive impairment, it primarily evaluates motor speed, psychomotor function, and memory recall [59]. In contrast, the WCST specifically assesses executive domains such as cognitive flexibility, set-shifting, and problem-solving ability [60]. Therefore, the lack of significant differences in IHDS scores between WCST-defined executive-impaired and unimpaired groups may reflect the distinct cognitive domains assessed by these instruments. Previous studies have shown that the IHDS performs better in identifying more advanced forms of HAND, particularly HIV-associated dementia, but is less sensitive for detecting milder or domain-specific impairment such as asymptomatic neurocognitive impairment and mild neurocognitive disorder [35, 36]. In our cohort, the relatively high proportion of participants with IHDS scores ≤10 further suggests that this cutoff may have limited utility for distinguishing subtle executive dysfunction in treated populations in the cART era. Therefore, while the IHDS remains a useful screening tool in resource-limited settings, more comprehensive or domain-specific neuropsychological assessments may be required for accurate detection of early or mild cognitive impairment in people living with HIV.

Taken together, the lack of significant differences in AD8 and IHDS scores between executive-impaired and unimpaired groups suggests that WCST-based phenotyping may capture subtle, domain-specific executive dysfunction not readily detected by brief global screening tools.

Three clinical and 3 laboratory variables are identified in our study for predicting executive phenotype, including years since HIV diagnosis, BMI classification (BMI < 27 vs BMI ≥ 27), nadir CD4 levels (CD4 ≥ 200 vs CD4 < 200), TIMP-1, TIMP-2, and TIMP-4. Clinical factors, including longer years since HIV diagnosis, and nadir CD4 < 200, were reported in previous research that are associated with poor cognitive function [12–14], which is also identified in our study for predicting executive impairment. Body weight is a relatively complex issue with neurocognitive impairment. In a previous study, McCutchan JA et al found a protective effect of increased BMI on an overall global measure of neurocognitive impairment [13]. But more recently, Olafor CN et al found obesity, rather than overweight, may contribute to cognitive processing speed deficits in adults with HIV [61]. Jumare J et al showed that neurocognitive impairment among patients with HIV-1 was more prevalent in overweight/obese individuals [62]. Rubin LH et al showed that BMI was inversely associated with motor function in HIV men [63]. In our study, obesity (BMI ≥ 27) similarly emerged as a predictor of executive impairment, suggesting that excess adiposity may exacerbate neurocognitive vulnerability in chronic HIV infection, potentially through metabolic dysregulation, systemic inflammation, or cerebrovascular mechanisms. To assess the relationships among the included variables, we evaluated multicollinearity using VIF, and all variables demonstrated low collinearity (VIF < 2; HIV duration: 1.03; BMI: 1.02; TIMP-1: 1.04; TIMP-2: 1.03; TIMP-4: 1.04; CD4: 1.02), indicating that the variables contributed independently to the model. In addition, SHAP analysis provided insight into the relative contribution of each variable, with TIMP-1 and duration of HIV infection demonstrating greater influence on model predictions, suggesting greater relative importance in predicting executive impairment. In addition to statistical significance, effect sizes were estimated to further characterize the magnitude of the observed associations. Variables including TIMP-1 (Cohen's *d* = 0.70), TIMP-2 (*d* = 0.43), TIMP-4 (*d* = 0.54), duration of HIV infection (*d* = 0.58), BMI (*d* = 0.64), and nadir CD4 count (*d* = 0.46) demonstrated varying effect sizes across predictors. Although several predictors reached statistical significance, their individual discriminative performance remained limited, suggesting that executive dysfunction in people living with HIV is likely multifactorial and not adequately explained by any single variable. These findings further support the use of combined predictive models integrating both clinical and biomarker data.

In this study, we applied machine-learning algorithms to predict the executive function impairment phenotype among middle-aged individuals living with HIV, using an integrated set of clinical and laboratory variables. Among single predictors, TIMP-1 exhibited the strongest discriminative performance (AUC = 0.76 by XGBoost), whereas conventional clinical indicators such as BMI and nadir CD4 count showed limited predictive ability (AUC ≈ 0.63). These findings underscore that no single biomarker or clinical factor can fully capture the complexity of executive dysfunction, reflecting the multifactorial pathogenesis of HAND. The substantial improvement observed when multiple variables were combined (AUC up to 0.89 with XGBoost) suggests that integrating biological and clinical data enhances predictive power by encompassing the diverse mechanisms contributing to executive function decline in HIV. Among the algorithms evaluated, XGBoost consistently achieved the highest accuracy, with a cross-validated mean AUC of 0.77, indicating good model robustness and generalizability. Collectively, these results highlight the potential of multiparameter, machine learning–based approaches to facilitate early identification and risk stratification of individuals with executive dysfunction. Given that executive impairment may be modifiable, this approach could ultimately support the development of precision tools for early detection and targeted neuropsychological evaluation in people living with HIV.

TIMP-1 is traditionally regarded as a key regulator of extracellular matrix turnover through its inhibition of MMPs. Experimental work by Tan et al [24] demonstrated that exogenous TIMP-1 confers robust neuroprotection by markedly reducing excitotoxic neuronal death, suggesting a direct role in maintaining neuronal integrity under pathological stress. Clinically, reduced TIMP-1 levels have been observed in patients with Alzheimer's disease and in elderly individuals at increased risk for prodromal dementia, implying that diminished TIMP-1 activity may contribute to neurodegenerative vulnerability [64]. Nevertheless, the precise function of TIMP-1 within the CNS remains uncertain. Growing evidence indicates that its actions extend beyond matrix regulation. TIMP-1 is now recognized as a multifunctional cytokine with context-dependent effects on immune and inflammatory signaling. Elevated circulating TIMP-1 concentrations have been documented across—as both a neuroprotective and proinflammatory mediator—and underscore the need to delineate the conditions under which TIMP-1 exerts beneficial versus detrimental effects on CNS integrity. The prominent predictive value of TIMP-1 in our model may reflect this dual biological role: elevated levels could represent a compensatory neuroprotective response to neuronal stress or, conversely, an indicator of chronic neuroinflammatory activity contributing to executive dysfunction.

It is also important to consider that the biomarkers in this study were measured in plasma rather than in cerebrospinal fluid or brain tissue. While CNS-derived biomarkers may more directly reflect neuroinflammatory processes, plasma biomarkers offer a more accessible and clinically feasible approach. Previous studies have demonstrated that circulating cytokines and inflammatory markers are associated with neurocognitive performance in people living with HIV, supporting the relevance of peripheral measurements. Nevertheless, plasma biomarkers may only partially reflect CNS-specific pathology, and their interpretation should be made with caution [65, 66].

This study has several limitations that should be acknowledged. First, in this study, executive function was assessed using the WCST as a representative domain, while other cognitive domains were not systematically evaluated. In addition, WCST performance was standardized using general population norms, which may not fully account for HIV-specific factors. Second, because a comprehensive neuropsychological evaluation is time-consuming, only 169 participants were included in the present cohort. A larger sample size would enhance the statistical power and improve the stability and generalizability of the prediction model. Third, although numerous biomarkers have been implicated in cognitive impairment among people living with HIV, our analysis focused specifically on MMPs and their tissue inhibitors (TIMPs), which represent a well-characterized but limited subset of potential candidates. In addition, plasma biomarkers may not fully reflect CNS-specific processes. Future studies incorporating a broader range of neuroinflammatory and neuronal injury markers may provide a more comprehensive understanding of the underlying mechanisms. Finally, the absence of external validation limits the immediate generalizability of our findings; validation in independent cohorts is warranted to confirm the robustness and clinical applicability of the proposed predictive model.

From a clinical perspective, our findings suggest a potential pathway for integrating data-driven approaches into the early identification of neurocognitive impairment in people living with HIV. Given that comprehensive neuropsychological assessments are resource-intensive and not routinely feasible in many clinical settings, a predictive model incorporating clinical variables and selected laboratory biomarkers may serve as a screening or triage tool to identify individuals at higher risk of executive dysfunction. Such an approach could facilitate targeted referral for formal neuropsychological evaluation and potentially enable earlier intervention strategies. While some biomarkers included in this study are not currently part of routine clinical testing, the model highlights biologically relevant factors that may inform future biomarker assay development and clinical implementation. Although the model requires external validation prior to clinical implementation, it represents an important step toward personalized risk stratification and precision medicine in the management of HAND.

## CONCLUSIONS

In this cohort of middle-aged Taiwanese individuals living with HIV, the occurrence of executive function impairment was about 9.5%, indicating that cognitive deficits continue to be prevalent even in the modern cART era. This notable burden highlights that achieving viral suppression alone does not completely eliminate the risk of neurocognitive complications. Considering the complex causes of HAND, it is crucial to develop predictive models for the early identification of at-risk individuals. Our results suggest that using machine learning to combine clinical and biological data could help in promptly identifying patients who may benefit from thorough neuropsychological assessment and early interventions to maintain cognitive health.

## Supplementary Material

ofag335_Supplementary_Data

## Supplementary Data

Supplementary materials are available at *Open Forum Infectious Diseases* online. Consisting of data provided by the authors to benefit the reader, the posted materials are not copyedited and are the sole responsibility of the authors, so questions or comments should be addressed to the corresponding author.
