# Supplementary material for: Machine-Learning Approach to Identify Tissue Inhibitors of Metalloproteinases (TIMP) and Clinical Variables Predicting Executive Phenotypes in HIV-infected Adults
Source: Open Forum Infect Dis. 2026 Jun 3;13(6):ofag335. doi: 10.1093/ofid/ofag335 (PMC13247526; doi:10.1093/ofid/ofag335)
Supplement: ofag335_Supplementary_Data [file ofag335_supplementary_data.docx]

**Supplementary Table 1** Raw scores and adjusted z-scores of WCST measures in executive-impaired and executive-unimpaired groups

|  | Unimpaired  (N=153) | Impaired  (N=16) |
| --- | --- | --- |
| Variables | mean ± SD | mean ± SD |
| Perseverative Responses, raw score | 24.8 ± 13.7 | 105.3 ± 21.2 |
| z-score | -0.11 ± 0.71 | 4.15 ± 1.15 |
| Total Errors, raw score | 42.4 ± 19.2 | 86.3 ± 12.5 |
| z-score | 0.06 ± 0.93 | 2.22 ± 0.62 |
| Perseverative Errors, raw score | 22.1 ± 11.2 | 80.7 ± 14.4 |
| z-score | -0.43 ± 0.58 | 2.60 ± 0.79 |
| Conceptual level responses, raw score | 56.2 ± 21.8 | 13.9 ± 12.1 |
| z score* | 0.12 ± 1.00 | 2.06 ± 0.57 |

Adjusted z-scores were calculated using locally derived normative data corrected for age, sex, and education. Higher z-scores indicate poorer executive performance.

* The direction of the z-score for this variable was reversed to maintain consistency in interpretation across WCST measures.
